# Multi-Access Channel Based on Quantum Detection in Wireless Optical Communication

**DOI:** 10.3390/e24081044

**Published:** 2022-07-29

**Authors:** Wenbin Yu, Fei Chen, Zeyu Xu, Yifan Zhang, Alex X. Liu, Chengjun Zhang

**Affiliations:** 1 Jiangsu Collaborative Innovation Center of Atmospheric Environment and Equipment Technology (CICAEET), Nanjing University of Information Science and Technology, Nanjing 210044, China; ywb@nuist.edu.cn; 2 School of Computer Science, Nanjing University of Information Science and Technology, Nanjing 210044, China; 20211221056@nuist.edu.cn (F.C.); 20211249352@nuist.edu.cn (Z.X.); zhangyifan0228@126.com (Y.Z.); 3 Engineering Research Center of Digital Forensics, Ministry of Education, Nanjing University of Information Science and Technology, Nanjing 210044, China; 4 Jiangsu Engineering Center of Network Monitoring, Nanjing University of Information Science and Technology, Nanjing 210044, China; 5 Department of Computer Science and Technology, Qilu University of Technology, Jinan 250353, China; alexliu360@gmail.com

**Keywords:** multi-access, wireless optical communication, multi-user detection, quantum measurement, quantum detection

## Abstract

In this paper, we propose a novel multi-user access in wireless optical communication based on the quantum detection of the coherent state. In this case, the coherent states are used as the signal carrier and a technique of quantum detection is applied to distinguish between signals from different users. To accomplish this task, two main quantum measurement methods are introduced; one is minimum error discrimination (MED), and the other is unambiguous state discrimination (USD). The theoretical derivation implies that the two methods can both distinguish between the signals from different users efficiently when the average photon number is large enough. Typically, the numerical result shows that in the two-user case, the channel capacity will approach the theoretical maximum limit when the average photon number is greater than 2.5 for MED and 5 for USD in the absence of noise. The MED gains more channel capacity than the USD at the same average photon number. However, the USD wins the error-correction scene with its free-error capability. Furthermore, the detection error probability and channel capacity for the USD with the thermal noise are examined. The result shows that increasing the signal average photon number can continue the USD’s advantage of error-free detection even if in the presence of thermal noise. In addition, compared with non-orthogonal multiple access (NOMA), the bit error rate (BER) against signal-to-noise rate (SNR) performance of USD has been improved.

## 1. Introduction

Wireless optical communication (WOC), a high-speed wireless communication technology, has been widely studied in recent years [1,2,3]. It has the advantages of fast deployment, high bandwidth quality, and high security [4,5]. More recent works delve into the system level by deploying cells of multi-point-to-point or multi-point-to-multi-point communication. The related multiple-input multiple-output (MIMO) principle has been investigated [6,7,8]. Usually, multiple access techniques include orthogonal multiple access (OMA) and non-orthogonal multiple access (NOMA). OMA is widely used and analyzed, such as for time division multiple access (TDMA), frequency division multiple access (FDMA) and code division multiple access (CDMA). In the system of [9], the authors proposed an optical CDMA (OCDMA) network. For OCDMA, optical orthogonal codes (OOCs) can be used to implement [10]. In the work of [11], TDMA of WOC is analyzed. Moreover, [12] suggests that NOMA is more suitable for WOC, especially in strong turbulence channels. Some recent work, such as the filter-enhanced scheme proposed in [13], shows promising solutions to providing high-speed spatially modulated optical wireless communication for multiple users.

On the other hand, as we know, the birth of quantum information science at the end of the last century is called a revolution in the world of science and technology. Since the beginning of the 21st century, quantum information science has been developing rapidly, and scholars from physics, computer science, communication electronics and other industries have joined the wave of research in the field of quantum information science, producing many remarkable research results in areas such as quantum communication, quantum computing, quantum control and so on. Quantum measurement is the cornerstone of the whole quantum information science building, and the detection of different quantum-state signals involves the basic principles of quantum measurement, especially in the WOC based on the coherent state, and the value of applying the latest quantum measurement research results cannot be overstated.

There are two complementary approaches to differentiating between non-orthogonal quantum states. The first one is minimum error discrimination (MED). This method aims to find the measurement of minimum error probability to identify different quantum states [14]. The second approach is called unambiguous state discrimination (USD). It introduces a perfect quantum-state-discrimination using uncertain results [15,16], which aims to maximize the probability of obtaining a definite result. However, the imperfection of reality makes the ideal error-free USD impossible. Therefore, real-world USD becomes a measurement strategy between MED and ideal USD, which contains some errors in these deterministic results even though the deterministic results of ideal USD can be obtained. Although traditional techniques for measuring coherent states based on post-selection exist, of course they obtain results with errors and uncertainties, and they do not improve the performance of standard quantum bounds [14,17]. However, compared to the many important advances made in the field of MED [14,18,19,20,21,22,23,24], the work on the study of non-orthogonal coherent-state USD is more limited [25,26,27]. Research on the experimental side has also been limited to performing USD for two coherent states [28], and the [29] introduced optimal USD for two matter quantum bits achieved using generalized measurements and showed that they were able to outperform standard projection measurements. The work in [30] gives a physical implementation of USD measurements of quadratic coherent states in reality. The feasibility of implementing USD measurements of multiple non-orthogonal coherent states and whether any ideal measurement that reaches the standard quantum bounds can be performed using the existing imperfect realistic conditions is an open question. A similar attempt to do so was made in [31] but proved to be unsuccessful [32].

Thanks to the development of quantum information, unlike the OMA and NOMA techniques, this paper attempts to provide a solution for multi-access in WOC from the perspective of quantum measurement. Unlike the conventional approach, we consider using symmetric coherent-state signals in the complex plane as carriers for multi-user WOCs. By using quantum detection, multi-access channel can be adapted to communication situations with lower optical signal power. It is well-suited for free-space optical communication, especially in deep space optical communication where the received signal power is highly restricted [33]. We let each user correspond to a pair of coherent-state signals, thus constructing a single-bit multi-access channel. This will be detailed in Section 2.2, and by applying MED and USD, we can obtain the optimal quantum detection and error-free detection of the coherent-state signals, respectively. These are placed in Section 2.3. Section 2.4 gives a typical example of two-user channel to facilitate our calculation of the channel capacity values. Further, we study the case of USD with thermal noise in Section 2.5 to evaluate the impact on error-free detection. Specific numerical experimental results are given in Section 3 to demonstrate the effectiveness of our approach.

## 2. Multi-Access Channel and Quantum Detection

### 2.1. Notation and Symbol Definitions

To facilitate reading and understanding of the subsequent chapters, we set up Table 1 here to elaborate on the definitions of symbols and marks in the text.

### 2.2. Multi-Access Channel Based on Symmetrical Coherent States

**Figure 1:**
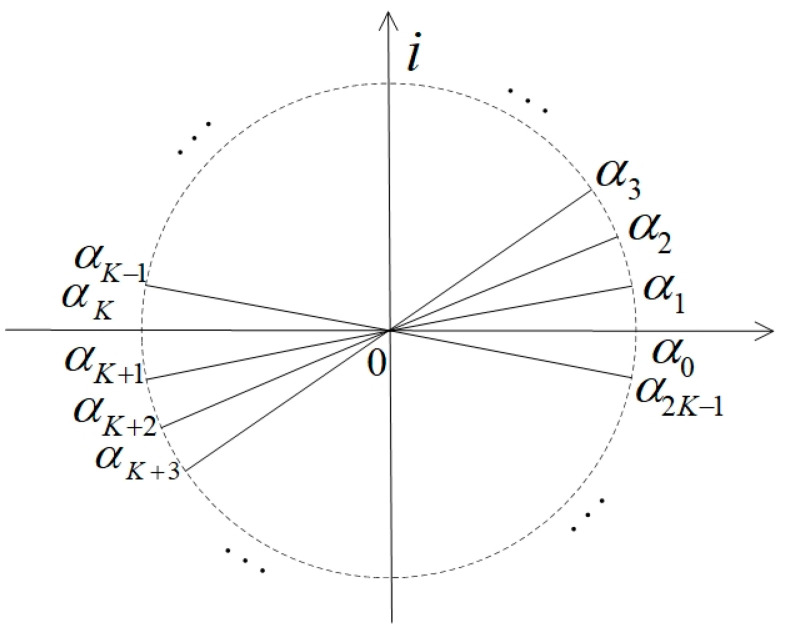
2K complex numbers symmetric to the origin of the complex plane.

Theoretically, the complex envelope α of the coherent-state signal can take on any value from the complex plane. Therefore, the following situation is considered here: assuming that α is taken as {0,1,…,2K−1}, there are 2K symmetric complex numbers with different arguments and the same modulus. The specific description is shown in Figure 1.

In Figure 1, $\alpha_{j}=\left|\alpha \right|e^{\frac{\pi ji}{K}}$; all plural numbers $\left\{\alpha_{j}\right\}$ have a modulus equal to |α|. Here,

(1) $$\left|\alpha_{j}\right\rangle =e^{-\frac{1}{2}{\left|\alpha \right|}^{2}}\sum_{n=0}^{\infty }\frac{\alpha_{j}{}^{n}}{\sqrt{n!}}\left|n\right\rangle ,\left(j\in \left\{0,1,\cdots 2K-1\right\}\right).$$

It can be seen from Figure 1 that α, which is symmetrical with respect to the origin of the complex plane, has an opposite −α. It appears K pairs of α and −α in total.

**Figure 2:**
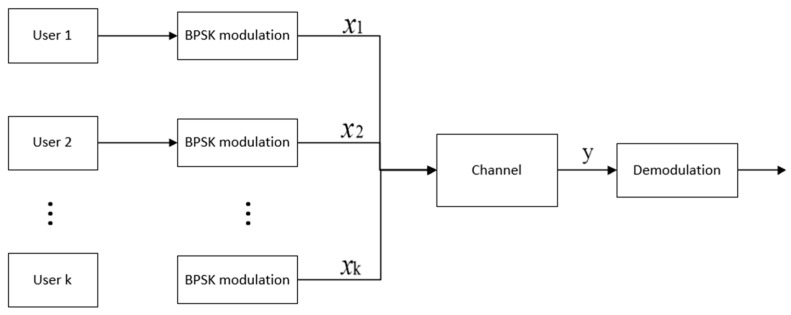
Multi-access channel based on symmetrical coherent-state signals.

According to the above analysis, we propose a multi-access channel model based on symmetric coherent-state signals. In order to facilitate the unification of the formula indicators, we assume that the number of users ranges from 0 to *K* − 1, with a total of K users. If 2K coherent states are assigned to K users, the jth user is required to enjoy a pair of coherent states $\left\{\left|\alpha_{j}\right\rangle ,\left|\alpha_{j+K}\right\rangle \right\}$, as shown in Figure 1, where j∈{0,1,⋯K−1} and satisfy $\left|\alpha_{j+K}\right\rangle =\left|-\alpha_{j}\right\rangle$. Each user adopts the BPSK method to modulate the transmitted information to the coherent state $\left\{\left|\alpha_{j}\right\rangle ,\left|\alpha_{j+K}\right\rangle \right\}$, as shown in Figure 2.

The signal of the user shown in Figure 2 has the following prior assumptions. Where

(2) $$\mathrm{User}:\;j\{\begin{array}{cc} H_{0}^{j} & \left|\alpha_{j}\right\rangle \\ H_{1}^{j} & |\alpha_{j+K}\rangle \end{array},\;\left(j=0,1,\cdots ,K-1\right).$$

For an ideal quantum multi-access channel, when the signal states sent by different users are all orthogonal, there is no inter-user interference. For coherent-state multi-access channels, since the coherent states are always non-orthogonal, inter-user interference will always exist. Therefore, the average inter-user interference can be reflected by the degree of overlap of the coherent-state signals of different users, that is

(3) $$r=\frac{1}{4K\left(K-1\right)}\sum_{\begin{array}{l} j,j^{\prime }=0,j^{\prime }\neq j\\ j^{\prime }\neq \left(j+K\right)mod2K \end{array}}^{2K-1}{\left|\langle \alpha_{j^{\prime }}|\alpha_{j}\rangle \right|}^{2}=\frac{1}{2\left(K-1\right)}\sum_{\begin{array}{l} j=1\\ j\neq K \end{array}}^{2K-1}exp\left(-{\left|\alpha \left(1-e^{\frac{\pi ji}{K}}\right)\right|}^{2}\right).$$

The value of r is between 0 and 1. The closer to 0, the smaller the interference, and the closer to 1, the stronger the interference.

**Figure 3:**
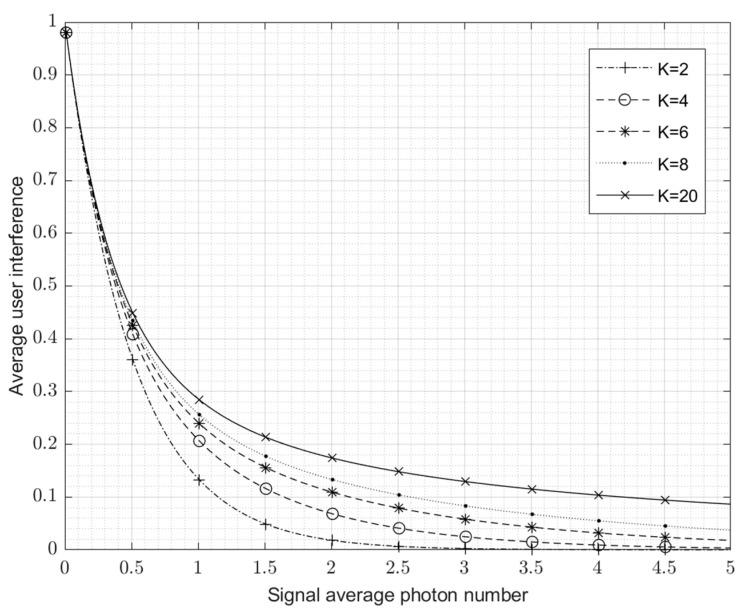
Average user interference, r, for multi-user coherent-state signals.

Figure 3 shows the average inter-user interference curve when the number of users, K, changes from 2 to 20. It implies that if the average photon number is large enough, the inter-code interference between users can be negligibly small even if the number of users is large.

### 2.3. Multi-User Detection Based on Quantum Measurement

The task of multi-user detection requires us to distinguish between different signals from different users. For this, there are many existing quantum multi-user detection methods available, such as optimal Bayesian criterion detection and least mean square detection. As far as the symmetric coherent states in the model are concerned, under the condition of equal prior probability, the least mean square detection is consistent with the optimal Bayesian criterion detection. In the physical sense, they are all MED measurements using orthogonal projection measurements. Therefore, after the measurement, the probability of an inter-user detection error is

(4) $$P_{e}^{\mathrm{MED}}=\sum_{j=0}^{K-1}\sum_{\begin{array}{l} k=0,k\neq j\\ k\neq j+K \end{array}}^{2K-1}\left[P\left(k|H_{0}^{j}\right)P\left(H_{0}^{j}\right)+P\left(k|H_{1}^{j}\right)P\left(H_{1}^{j}\right)\right].$$

Because the prior probability of the signal set sent by all users is assumed to be equal, so

(5) $$P\left(H_{0}^{j}\right)=P\left(H_{1}^{j}\right)=\frac{1}{2K}.$$

The above equation is simplified to

(6) $$P_{e}^{\mathrm{MED}}=\frac{1}{2K}\sum_{j=0}^{K-1}\sum_{\begin{array}{l} k=0,k\neq j\\ k\neq j+K \end{array}}^{2K-1}\left[P\left(k|H_{0}^{j}\right)+P\left(k|H_{1}^{j}\right)\right].$$

The ultimate purpose of the above detection method is to minimize the average detection error probability. However, for each individual detection process, there is always a certain probability of error in detection, resulting in ambiguous results. Therefore, these detection methods are vague detections.

On the other hand, let us say we take the USD measurement. Then, it is necessary to apply the generalized quantum measurement principle to detect all user signal sets, and to construct a common POVM operator uniformly. Its purpose is to obtain clear test results.

Below, we give the construction of the POVM operator measured using USD. First, according to the coherent state’s definition, the following properties are obtained.

(7a) $$\left|\alpha_{j+1}\right\rangle =U\left|\alpha_{j}\right\rangle =U^{j}\left|\alpha_{0}\right\rangle ,$$

(7b) $$\left|\alpha_{0}\right\rangle =U\left|\alpha_{2K-1}\right\rangle ,$$

(7c) $$U^{2K}=I.$$

It can be verified that the form of the unitary operator in the above equation is

(8) $$U=P_{H}e^{\frac{\pi i\hat{n}}{K}}P_{H}.$$

Here, $\hat{n}=a^{+}a$ is the photon number operator, and its eigenstate is the photon number state.

According to the forms of Equations (7) and (8), we further construct the eigenstates of the operator U and use them as a set of canonical orthonormal bases of the subspace H, namely

(9) $$U=\sum_{k=0}^{2K-1}e^{\frac{k\pi i}{K}}\left|\gamma_{k}\right\rangle \left\langle \gamma_{k}\right|.$$

where $\langle \gamma_{k}|\gamma_{k^{\prime }}\rangle =\delta_{kk^{\prime }}$; it is easy to know that $\left|\gamma_{k}\right\rangle$ is orthonormal. Since there are linearly independent coherent states between $\left|\alpha_{j}\right\rangle$, we have

(10) $$\left|\alpha_{j}\right\rangle =\sum_{k=0}^{2K-1}c_{k}e^{\frac{\pi kji}{K}}\left|\gamma_{k}\right\rangle .$$

The corresponding reciprocal state is given by

(11) $$\left|\alpha_{j}^{⊥}\right\rangle =Z^{-\frac{1}{2}}\sum_{k=0}^{2K-1}c_{K}^{*-1}e^{\frac{\pi kji}{K}}\left|\gamma_{k}\right\rangle .$$

In it,

(12) $$Z=\sum_{k=0}^{2K-1}{\left|c_{k}\right|}^{-2}.$$

According to the symmetry of the signal state, the POVM operators $\left\{E_{0},\cdots ,E_{2K-1},E_{F}\right\}$ with the number of 2K+1 can be given by the following equations.

(13a) $$E_{j}=\frac{P_{j}}{4K^{2}}\sum_{k,k^{\prime }=0}^{2K-1}c_{k^{\prime }}^{*-1}c_{k}^{-1}e^{\frac{\pi ji\left(k-k^{\prime }\right)}{K}}\left|\gamma_{k^{\prime }}\right\rangle \left\langle \gamma_{k}\right|,\left(0\leq j\leq 2K-1\right),$$

(13b) $$E_{F}=I-\sum_{j=0}^{2K-1}E_{j}.$$

In Equation (13), $P_{j}$ represents the probability of obtaining result j after measurement when the measured state is $\left|\alpha_{j}\right\rangle$.

Considering the existence of noise in the channel, it is assumed that the transmitted signal is pure state $\left|\alpha_{j}\right\rangle$, and the pure state signal is correspondingly transformed into $\rho_{j}$ after passing through the channel ($\rho_{j}$ is the mixed-state density operator). Then, the jth user prepares the signal l in advance, and after the measurement, the probability of obtaining the result k is

(14) $$P\left(k|H_{l}^{j}\right)=tr\left(E_{k}\rho_{j+lK}\right).$$

In the above equation, k=0,1,⋯,2K, j=0,1,⋯,K−1, l∈{0,1}. Substituting Equation (14) into Equation (6), it can be concluded that under the premise of using the USD measurement, the probability of detection error between users is

(15) $$P_{e}^{\mathrm{USD}}=\frac{1}{2K}\sum_{j=0}^{K-1}\sum_{\begin{array}{c} k=0,k\neq j\\ k\neq j+K \end{array}}^{2K-1}\left[tr\left(E_{k}\left(\rho_{j}+\rho_{j+K}\right)\right)\right].$$

On the other hand, if the channel contains no noise background, the measurement results are transformed into

(16) $$P\left(k|H_{l}^{j}\right)=tr\left(E_{k}\left|\alpha_{j+lK}\right\rangle \left\langle \alpha_{j+lK}\right|\right).$$

This is easy to verify based on the nature of USD measurements:

(17) $$P\left(k|H_{l}^{j}\right)=0,\left(k\neq j+lK\right).$$

Combining the above equation and Equation (15), it can be inferred that the detection error probability between users without noise background is

(18) $$P_{e}^{\mathrm{USD}}=0.$$

From a theoretical point of view, this means that if the effects of noise are ignored, USD measurements can perfectly detect the signals sent by different users from multi-access channels. Accordingly, MED measurements can only vaguely detect the signals of different users because its orthogonal measurement mechanism will always maintain a certain detection error probability.

### 2.4. Two-User Channel Capacities Based on MED and USD

**Figure 4:**
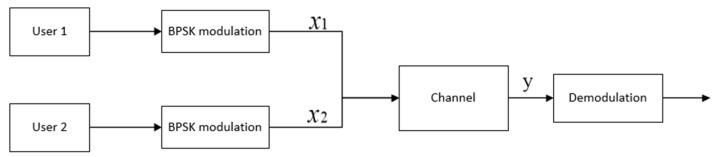
Two-user channel based on coherent-state signals.

Next, we take the two-user channel as the research object. The channel model of two users based on symmetrical coherent-state signals is shown in Figure 4.

Two users have a set of coherent-state signals modulated by BPSK, respectively,

$$\begin{array}{c} \mathrm{User}\;1:\;\{\begin{array}{cc} H_{0}^{0} & \left|\alpha_{0}\right\rangle =\left|\alpha \right\rangle \\ H_{1}^{0} & \left|\alpha_{2}\right\rangle =\left|-\alpha \right\rangle \end{array}\\ \mathrm{User}\;2:\;\{\begin{array}{cc} H_{0}^{1} & \left|\alpha_{1}\right\rangle =\left|i\alpha \right\rangle \\ H_{1}^{1} & \left|\alpha_{3}\right\rangle =\left|-i\alpha \right\rangle \end{array} \end{array}$$

According to the analysis in the previous section, from the conclusions of [34,35] about MED measurement, the measurement probability of the two-user MED measurement falling on each result can be calculated as follows:

(19) $$\begin{array}{c} P\left(k|H_{0}^{0}\right)=\frac{1}{4}\times {\left(\overset{3}{\underset{m,n=0}{\sum }}\frac{exp\left(\left(e^{\frac{\pi m}{2}i}-1\right){\left|\alpha \right|}^{2}+\frac{\pi m\left(\left(m-k\right)mod4\right)}{2}i\right)}{2\sqrt{1+e^{-{\left|\alpha \right|}^{2}+\left({\left|\alpha \right|}^{2}+\frac{\pi n}{2}\right)i}+e^{-2{\left|\alpha \right|}^{2}+\pi ni}+e^{-{\left|\alpha \right|}^{2}-\left({\left|\alpha \right|}^{2}-\frac{3\pi n}{2}\right)i}}}\right)}^{2},\\ \left(k\in \left\{0,1,2,3\right\}\right). \end{array}$$

The above equation represents the probability that user 1 transmits the signal state |α⟩, and the measurement result of the receiver is k. According to the prior probability equality and symmetry of the transmitted signal, the more general expression of measurement probability is deduced as

(20) $$P\left(k|H_{l}^{j}\right)=P\left(\left(k-j-2l\right)mod4|H_{0}^{0}\right).$$

Among it, k∈{0,1,2,3}, j,l∈{0,1}. Therefore, from Equations (6) and (20), it can be known that the inter-user detection error probability measured by the two-user MED is

(21) $$\begin{array}{c} P_{e}^{\mathrm{MED}\_2}=\frac{1}{4}\overset{1}{\underset{j=0}{\sum }}\overset{3}{\underset{\begin{array}{l} k=0,k\neq j\\ k\neq j+2 \end{array}}{\sum }}\left[P\left(k|H_{0}^{j}\right)+P\left(k|H_{1}^{j}\right)\right]\\ =P\left(1|H_{0}^{0}\right)+P\left(3|H_{0}^{0}\right). \end{array}$$

On the other hand, consider the result of Equation (18). Under equal conditions, perfect error-free detection between users can be achieved with ideal USD measurements. This shows that in the problem of two-user communication, USD measurement will have a better user discrimination performance than MED measurement.

As far as the channel capacity of the two-user communication is concerned, the MED measurement will theoretically obtain the maximum capacity of the coherent-state two-user channel. Here, finding the maximum channel capacity is equivalent to finding the Von Neumann Entropy of the coherent-state signal. The mixed state of the transmitted signal is

(22) $$\rho =\frac{1}{4}\sum_{j=0}^{3}\left|\alpha_{j}\right\rangle \left\langle \alpha_{j}\right|.$$

From Equation (22), using the MED measurement, the maximum capacity of the coherent-state two-user channel is found to be

(23) $$\begin{array}{l} \;\;\;\;\;\;\;\;\;\;\;\;\;\;\;\;\;\;\;C^{\mathrm{MED}\_2}=S\left(\rho \right)\\ =S\left(\frac{1}{4}\overset{3}{\underset{j=0}{\sum }}\left|\alpha_{j}\right\rangle \left\langle \alpha_{j}\right|\right)\\ =\frac{1}{4}\sum_{j=0}^{3}f_{j}\left(\alpha \right)\left(2-log\left(f_{j}\left(\alpha \right)\right)\right). \end{array}$$

In it,

(24) $$f_{j}\left(\alpha \right)=\left(1+e^{-{\left|\alpha \right|}^{2}+\left({\left|\alpha \right|}^{2}+\frac{\pi j}{2}\right)i}+e^{-2{\left|\alpha \right|}^{2}+\pi ji}+e^{-{\left|\alpha \right|}^{2}-\left({\left|\alpha \right|}^{2}-\frac{3\pi j}{2}\right)i}\right).$$

In addition, if the channel capacity of the coherent-state two-user channel for the USD case is theoretically investigated, it can be inferred that the maximum channel capacity that can be achieved by the USD measurement will be slightly lower than that of the MED measurement. To this end, the following will give the maximum channel capacity expression of the two-user USD measurement based on the result of the BPSK signal detection.

First, according to Equations (16) and (17), the probability that the USD measurement of two users falls on each result is

(25a) $$P\left(k|H_{l}^{j}\right)=0,\left(k\neq j+2l\right),$$

(25b) $$P\left(j+2l|H_{l}^{j}\right)=tr\left(E_{j+2l}\left|\alpha_{j+2l}\right\rangle \left\langle \alpha_{j+2l}\right|\right)=P_{D},$$

(25c) $$P\left(4|H_{l}^{j}\right)=1-P\left(j+2l|H_{l}^{j}\right).$$

where k∈{0,1,2,3}, j,l∈{0,1}, and $P_{D}$ is defined as

$$P_{D}=\frac{1}{4}\times min\left({∥|\phi_{0}\rangle ∥}^{2},{∥|\phi_{1}\rangle ∥}^{2},{∥|\phi_{2}\rangle ∥}^{2},{∥|\phi_{3}\rangle ∥}^{2}\right),$$

$$\begin{array}{c} ∥|\phi_{0}\rangle ∥=\sqrt{\left(\left\langle \alpha \right|+\left\langle i\alpha \right|+\left\langle -\alpha \right|+\langle -i\alpha |\right)\left(\left|\alpha \right\rangle +\left|i\alpha \right\rangle +\left|-\alpha \right\rangle +\left|-i\alpha \right\rangle \right)}=\\ 2\sqrt{1+e^{-{\left|\alpha \right|}^{2}+i{\left|\alpha \right|}^{2}}+e^{-2{\left|\alpha \right|}^{2}}+e^{-{\left|\alpha \right|}^{2}-i{\left|\alpha \right|}^{2}}},\\ ∥|\phi_{1}\rangle ∥=\sqrt{\left(\left\langle \alpha \right|-i\left\langle i\alpha \right|-\left\langle -\alpha \right|+i\left\langle -i\alpha \right|\right)\left(\left|\alpha \right\rangle +i\left|i\alpha \right\rangle -\left|-\alpha \right\rangle -i\left|-i\alpha \right\rangle \right)}=\\ 2\sqrt{1+ie^{-{\left|\alpha \right|}^{2}+i{\left|\alpha \right|}^{2}}-e^{-2{\left|\alpha \right|}^{2}}-ie^{-{\left|\alpha \right|}^{2}-i{\left|\alpha \right|}^{2}}},\\ ∥|\phi_{2}\rangle ∥=\sqrt{\left(\left\langle \alpha \right|-\left\langle i\alpha \right|+\left\langle -\alpha \right|-\left\langle -i\alpha \right|\right)\left(\left|\alpha \right\rangle -\left|i\alpha \right\rangle +\left|-\alpha \right\rangle -\left|-i\alpha \right\rangle \right)}=\\ 2\sqrt{1-e^{-{\left|\alpha \right|}^{2}+i{\left|\alpha \right|}^{2}}+e^{-2{\left|\alpha \right|}^{2}}-e^{-{\left|\alpha \right|}^{2}-i{\left|\alpha \right|}^{2}}},\\ ∥|\phi_{3}\rangle ∥=\sqrt{\left(\left\langle \alpha \right|+i\left\langle i\alpha \right|-\left\langle -\alpha \right|-i\left\langle -i\alpha \right|\right)\left(\left|\alpha \right\rangle -i\left|i\alpha \right\rangle -\left|-\alpha \right\rangle +i\left|-i\alpha \right\rangle \right)}=\\ 2\sqrt{1-ie^{-{\left|\alpha \right|}^{2}+i{\left|\alpha \right|}^{2}}-e^{-2{\left|\alpha \right|}^{2}}+ie^{-{\left|\alpha \right|}^{2}-i{\left|\alpha \right|}^{2}}}. \end{array}$$

Secondly, it is assumed that $X_{0}$ represents the input signal source of user 0, $X_{1}$ represents the input signal source of user 1, and Y represents the output signal source of the receiver. $X_{0}$ takes the value of $\left\{x_{k}^{0}|k\in \left\{0,1\right\}\right\}$, and $X_{1}$ takes the value of $\left\{x_{l}^{1}|l\in \left\{0,1\right\}\right\}$. Y takes the value of $\left\{y_{ij}|i,j\in \left\{0,1,2,3,4\right\}\right\}$ according to different measurement results. The transition probability of a coherent-state two-user channel measured by USD is

(26a) $$P\left(y_{01}|x_{0}^{0}x_{0}^{1}\right)=P\left(y_{03}|x_{0}^{0}x_{1}^{1}\right)=P\left(y_{21}|x_{1}^{0}x_{0}^{1}\right)=P\left(y_{23}|x_{1}^{0}x_{1}^{1}\right)=P_{D}^{2},$$

(26b) $$P\left(y_{41}|x_{k}^{0}x_{0}^{1}\right)=P\left(y_{43}|x_{k}^{0}x_{1}^{1}\right)=P\left(y_{04}|x_{0}^{0}x_{l}^{1}\right)=P\left(y_{24}|x_{1}^{0}x_{l}^{1}\right)=P_{D}\left(1-P_{D}\right),$$

(26c) $$P\left(y_{44}|x_{k}^{0}x_{l}^{1}\right)={\left(1-P_{D}\right)}^{2}.$$

For the remaining cases, the transition probability is always 0.

Therefore, according to Equation (26), it can be deduced that the maximum channel capacity of the coherent-state two-user channel measured by USD is

(27) $$\begin{array}{l} \;C^{\mathrm{USD}\_2}=I\left(X_{0}X_{1};Y\right)\\ =H\left(Y\right)-H\left(Y|X_{0}X_{1}\right)\\ =-\left(P_{D}^{2}log\frac{P_{D}^{2}}{4}+2P_{D}\left(1-P_{D}\right)log\frac{P_{D}\left(1-P_{D}\right)}{2}+{\left(1-P_{D}\right)}^{2}log{\left(1-P_{D}\right)}^{2}\right)\\ \;\;\;\;+P_{D}^{2}logP_{D}^{2}+2P_{D}\left(1-P_{D}\right)logP_{D}\left(1-P_{D}\right)\\ \;\;\;\;+{\left(1-P_{D}\right)}^{2}log{\left(1-P_{D}\right)}^{2}\\ =2P_{D}\\ =2\times min(1+ie^{-{\left|\alpha \right|}^{2}+i{\left|\alpha \right|}^{2}}-e^{-2{\left|\alpha \right|}^{2}}-ie^{-{\left|\alpha \right|}^{2}-i{\left|\alpha \right|}^{2}},1-e^{-{\left|\alpha \right|}^{2}+i{\left|\alpha \right|}^{2}}+e^{-2{\left|\alpha \right|}^{2}}\\ \;\;\;\;-e^{-{\left|\alpha \right|}^{2}-i{\left|\alpha \right|}^{2}},1-ie^{-{\left|\alpha \right|}^{2}+i{\left|\alpha \right|}^{2}}-e^{-2{\left|\alpha \right|}^{2}}+ie^{-{\left|\alpha \right|}^{2}-i{\left|\alpha \right|}^{2}}). \end{array}$$

### 2.5. Capacity of Thermal Noise Channel

In addition, the influence of thermal field noise on two-user detection with USD is further considered. According to the results of the detection error probability of the multi-access channel deduced in Section 2.2, the detection error probability expression between the two users with the thermal noise can be derived. We can obtain

(28) $$\begin{array}{cc} P_{e}^{\mathrm{USD}\_2} & =P\left(1|H_{0}^{0}\right)+P\left(3|H_{0}^{0}\right)\\ & =\frac{P_{D}Z}{16\pi N}\int exp\left(\frac{-{\left|\mu -\alpha \right|}^{2}}{N}\right)\left({\left|\langle \mu |i\alpha^{⊥}\rangle \right|}^{2}+{\left|\langle \mu |-i\alpha^{⊥}\rangle \right|}^{2}\right)d^{2}\mu \\ & =\frac{P_{D}}{16\pi N}\int \sum_{j,k=0}^{3}\left(A_{\left(j+3\right)mod4}A_{\left(k+3\right)mod4}^{*}+A_{\left(j+1\right)mod4}A_{\left(k+1\right)mod4}^{*}\right)\times \\ & exp\left(\frac{-{\left|\mu -\alpha \right|}^{2}}{N}-{\left|\mu \right|}^{2}-{\left|\alpha \right|}^{2}+e^{\frac{\pi ji}{2}}\mu^{*}\alpha +e^{-\frac{\pi ki}{2}}\alpha^{*}\mu \right)d^{2}\mu . \end{array}$$

Here, $A_{j}$ is

$$\begin{array}{c} A_{0}=\frac{4}{{∥|\phi_{0}\rangle ∥}^{2}}+\frac{4}{{∥|\phi_{1}\rangle ∥}^{2}}+\frac{4}{{∥|\phi_{2}\rangle ∥}^{2}}+\frac{4}{{∥|\phi_{3}\rangle ∥}^{2}},\\ A_{1}=\frac{4}{{∥|\phi_{0}\rangle ∥}^{2}}+\frac{4i}{{∥|\phi_{1}\rangle ∥}^{2}}-\frac{4}{{∥|\phi_{2}\rangle ∥}^{2}}-\frac{4i}{{∥|\phi_{3}\rangle ∥}^{2}},\\ A_{2}=\frac{4}{{∥|\phi_{0}\rangle ∥}^{2}}-\frac{4}{{∥|\phi_{1}\rangle ∥}^{2}}+\frac{4}{{∥|\phi_{2}\rangle ∥}^{2}}-\frac{4}{{∥|\phi_{3}\rangle ∥}^{2}},\\ A_{3}=\frac{4}{{∥|\phi_{0}\rangle ∥}^{2}}-\frac{4i}{{∥|\phi_{1}\rangle ∥}^{2}}-\frac{4}{{∥|\phi_{2}\rangle ∥}^{2}}+\frac{4i}{{∥|\phi_{3}\rangle ∥}^{2}}. \end{array}$$

From Equation (28), we can see that the factors affecting the error probability are signal power and noise power. Although the equation cannot be explicitly expressed in terms of the SNR, we can use $SNR={\left|\mu \right|}^{2}/N$. This facilitates our discussion of BER-SNR performance in Section 4.

Further, the channel capacity of the coherent-state two-user channel measured by USD under the background of thermal field noise is

(29) $$\begin{array}{l} \;\;\;\;\;\;\;\;\;\;\;C_{N}^{\mathrm{USD}\_2}=I\left(X_{0}X_{1};Y\right)\\ =H\left(Y\right)-H\left(Y|X_{0}X_{1}\right)\\ =-\sum_{i,j=0}^{4}P\left(y_{ij}\right)log\left(P\left(y_{ij}\right)\right)+\\ \;\;\;\;\;\;\;\;\;\;\;\;\;\;\;\;\;\;\;\;\;\;\;\;\;\;\;\;\;\;\;\;\;\;\sum_{k,l=0}^{1}\sum_{i,j=0}^{4}P\left(x_{k}^{0}x_{l}^{1}y_{ij}\right)log\left(P\left(y_{ij}|x_{k}^{0}x_{l}^{1}\right)\right). \end{array}$$

Considering the expansion of the above equation to all detection results, we obtain

(30) $$\begin{array}{c} =\frac{1}{4}\sum_{i,j=0}^{4}\underset{\begin{array}{c} k\in \left\{0,1\right\}\\ l\in \left\{2,3\right\} \end{array}}{\sum }P\left(i|H_{k}\right)P\left(j|H_{l}\right)\left(2-log\left(\underset{\begin{array}{c} k\in \left\{0,1\right\}\\ l\in \left\{2,3\right\} \end{array}}{\sum }P\left(i|H_{k}\right)P\left(j|H_{l}\right)\right)\right)+\\ \sum_{i,j=0}^{4}P\left(i|H_{0}\right)P\left(j|H_{1}\right)logP\left(i|H_{0}\right)P\left(j|H_{1}\right). \end{array}$$

Here, $P\left(i|H_{k}\right)$ is

$$\begin{array}{c} P\left(0|H_{0}\right)=P\left(1|H_{1}\right)=P\left(2|H_{2}\right)=P\left(3|H_{3}\right)=\frac{P_{D}Z}{16\pi N}\int exp\left(\frac{-{\left|\mu -\alpha \right|}^{2}}{N}\right){\left|\langle \mu |\alpha^{⊥}\rangle \right|}^{2}d^{2}\mu \;\\ P\left(1|H_{0}\right)=P\left(2|H_{1}\right)=P\left(3|H_{2}\right)=P\left(0|H_{3}\right)=\frac{P_{D}Z}{16\pi N}\int exp\left(\frac{-{\left|\mu -\alpha \right|}^{2}}{N}\right){\left|\langle \mu |i\alpha^{⊥}\rangle \right|}^{2}d^{2}\mu ,\;\\ P\left(2|H_{0}\right)=P\left(3|H_{1}\right)=P\left(0|H_{2}\right)=P\left(1|H_{3}\right)=\frac{P_{D}Z}{16\pi N}\int exp\left(\frac{-{\left|\mu -\alpha \right|}^{2}}{N}\right){\left|\langle \mu |-\alpha^{⊥}\rangle \right|}^{2}d^{2}\mu ,\\ P\left(3|H_{0}\right)=P\left(0|H_{1}\right)=P\left(1|H_{2}\right)=P\left(2|H_{3}\right)=\frac{P_{D}Z}{16\pi N}\int exp\left(\frac{-{\left|\mu -\alpha \right|}^{2}}{N}\right){\left|\langle \mu |-i\alpha^{⊥}\rangle \right|}^{2}d^{2}\mu . \end{array}$$

where

$$\begin{array}{c} {\left|\left\langle \mu |\alpha^{⊥}\right\rangle \right|}^{2}=Z^{-1}\sum_{j,k=0}^{3}A_{j}A_{k}^{*}exp\left(-{\left|\mu \right|}^{2}-{\left|\alpha \right|}^{2}+e^{\frac{\pi ji}{2}}\mu^{*}\alpha +e^{-\frac{\pi ki}{2}}\alpha^{*}\mu \right),\\ {\left|\langle \mu |i\alpha^{⊥}\rangle \right|}^{2}=Z^{-1}\sum_{j,k=0}^{3}A_{\left(j+3\right)mod4}A_{\left(k+3\right)mod4}^{*}exp\left(-{\left|\mu \right|}^{2}-{\left|\alpha \right|}^{2}+e^{\frac{\pi ji}{2}}\mu^{*}\alpha +e^{-\frac{\pi ki}{2}}\alpha^{*}\mu \right),\\ {\left|\langle \mu |-\alpha^{⊥}\rangle \right|}^{2}=Z^{-1}\sum_{j,k=0}^{3}A_{\left(j+2\right)mod4}A_{\left(k+2\right)mod4}^{*}exp\left(-{\left|\mu \right|}^{2}-{\left|\alpha \right|}^{2}+e^{\frac{\pi ji}{2}}\mu^{*}\alpha +e^{-\frac{\pi ki}{2}}\alpha^{*}\mu \right),\\ {\left|\langle \mu |-i\alpha^{⊥}\rangle \right|}^{2}=Z^{-1}\sum_{j,k=0}^{3}A_{\left(j+1\right)mod4}A_{\left(k+1\right)mod4}^{*}exp\left(-{\left|\mu \right|}^{2}-{\left|\alpha \right|}^{2}+e^{\frac{\pi ji}{2}}\mu^{*}\alpha +e^{-\frac{\pi ki}{2}}\alpha^{*}\mu \right), \end{array}$$

$$Z=16\times \left({∥|\phi_{0}\rangle ∥}^{-2}+{∥|\phi_{1}\rangle ∥}^{-2}+{∥|\phi_{2}\rangle ∥}^{-2}+{∥|\phi_{3}\rangle ∥}^{-2}\right).$$

## 3. Results and Analysis

### 3.1. Setup for Numerical Results

To analyze the effect of quantum detection more intuitively, we need to perform numerical verification of the theoretical results. The settings for the numerical results for MED and USD are given here.

In Section 3.2, the experiments do not involve the effect of noise, so we examine the effect of signal power on the error performance and channel capacity. Without loss of generality, the average photon number is used to measure the strength of the optical signal power. Here, ${\left|\mu \right|}^{2}$ characterizes the average photon number of the signal and the output is the channel capacity. In Section 3.3, in addition to setting ${\left|\mu \right|}^{2}$, the experiment also considers the effect of thermal noise power on the channel performance. We let N characterize the average photon number of thermal noise and examine the output as the inter-user detection error probability and channel capacity. In these experiments, ${\left|\mu \right|}^{2}$ is taken in the range of 0.1 to 5 and N is taken in the range of 0.01 to 0.5. Other parameters, such as SNR, will be used in Section 4.2. The definitions of the symbols involved can be found in Table 1.

### 3.2. Numerical Result of Two-User Detection in Absence of Noise

Section 2.3 theoretically gives the effect of using ideal USD measurement to achieve error-free inter-user detection in a noise-free situation, while MED measurement under the same conditions can only give detection results with a certain error probability. 

In this section, numerical experiments are given to the above results to investigate the maximum channel capacity when the coherent-state signals of two users are measured by MED and USD without any noise background. As well as the inter-user detection error probability and the maximum channel capacity measured by USD in the presence of thermal noise will be presented in the next section.

**Figure 5:**
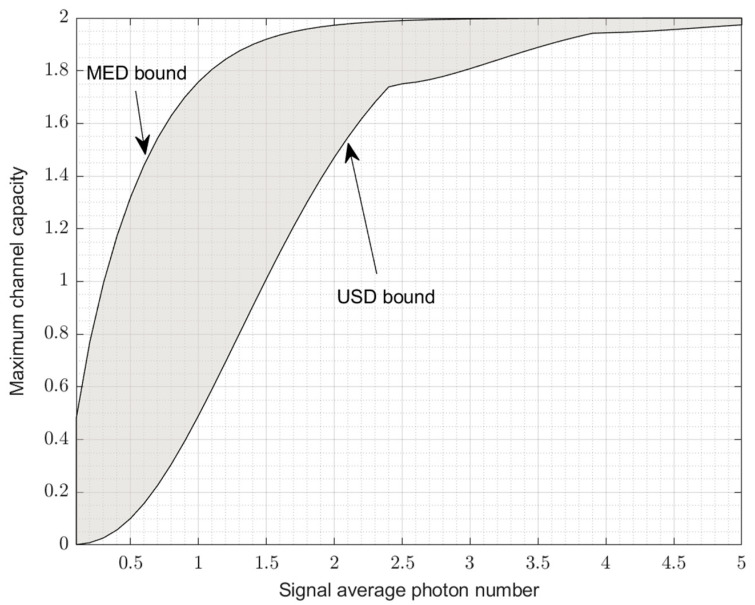
The maximum capacities of MED and USD for two-user channel, respectively.

Based on the calculation of Equations (23) and (27), Figure 5 depicts the maximum channel capacity curve measured by MED and USD, and the gray area between the two curves is the channel capacity area that is reachable by MED but not by USD. This shows that with regard to the multi-user detection problem, the USD measurement can obtain a better inter-user detection error probability (ideally no error) than the MED measurement, while the reachable area of the channel capacity is smaller than that of the MED measurement.

As can be seen from Figure 5, the difference between the two is more obvious in the part where the average photon number of the signal is small. The maximum channel capacity for single-bit two-user communication is 2. The MED already exceeds 1.8 at an average photon number of 1.5 and is close to 2 at an average photon number of 3. For the USD case, although its channel capacity is lower than that of the MED, it is close to 2 when the average photon number exceeds five. This is efficient for multi-user communication, as theoretically they only need less power for coherent-state signals.

On the other hand, as a theoretical limit of the channel capacity, it is practically difficult to reach, because there is always noise. In fact, for MED, Helstrom gives in [14] the theoretical limit of binary quantum detection, which is exactly the MED bound in Figure 5. Additionally, the actual receiver can overcome the effect of noise close to the performance of the Helstrom bound [36] when the signal power is large enough. So, the typical range of capacity is achievable up to the gray area close to the MED bound in Figure 5.

For USD, what we know is that the typical range of capacity should be reachable in the area below the USD bound in Figure 5. However, the exact details of how to do this are much more complicated because the case of USD under noise for a general quantum mixed state has not been sufficiently studied [31,32]. However, based on the symmetric coherent-state signal used in this paper, the noise problem of USD can be solved to some extent by using some mathematical tricks. This part will be developed in the next section.

### 3.3. USD Detection Performance in Presence of Thermal Noise

Unlike MED, where the impact of noise on MED only increases its average error probability and reduces its channel capacity, noise challenges USD’s advantage of having zero errors, so it is necessary to study how much thermal noise affects USD’s errors.

**Figure 6:**
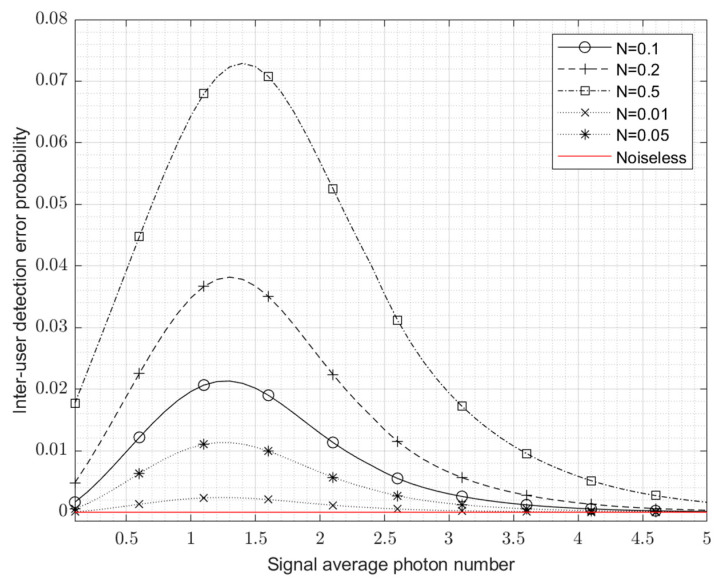
Detection error probability between two users of USD in the background of thermal noise.

Figure 6 plots the inter-user detection error probability for the average photon number of the thermal noise power N from 0.01 to 0.5, according to the calculation results of Equation (28). The red line in the figure indicates the noise-free case, where the error probability is 0, i.e., the effect of error-free discrimination of USD is achieved when the noise is absent.

As can be seen from Figure 6, when the noise average photon number increases, the inter-user detection error probability of USD also increases. A clear peak is formed between 1 and 1.5 of the signal average photon number. There are two reasons for this. On the one hand, with the increase in the signal power, the probability of detection errors between users tends to decrease; on the other hand, the USD measurement degenerated into a general quantum measurement due to the noise that cannot be ignored in relation to the signal. At this time, although there is a small detection error probability between users, the detection error probability of the BPSK signal owned by each user increases, but the overall detection performance decreases.

**Figure 7:**
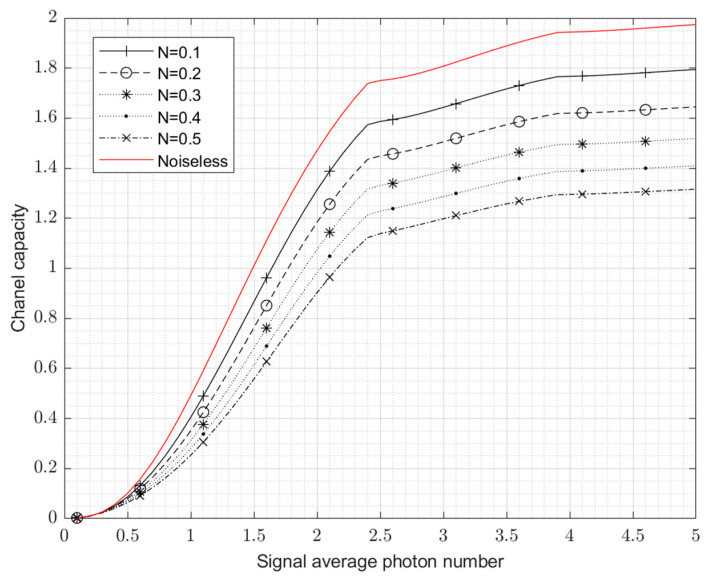
The maximum channel capacity of USD in the background of thermal noise (noise average photon number from 0.1 to 0.5).

Depending on the calculation result of Equation (30), Figure 7 depicts the USD’s channel capacity curve with the noise power varying uniformly from 0.1 to 0.5. The red line here indicates the channel capacity for the noiseless case, which is in fact consistent with the USD bound in Figure 5.

When the average photon number increases further beyond five, the degrading effect of noise on the USD measurements will gradually decrease, and USD will still obtain a low probability of detection error and a high channel capacity, as can be seen in Figure 6 and Figure 7. This means that after a certain value of signal power, e.g., an average photon number above five, increasing the SNR allows error-free detection to continue to be effective.

## 4. Discussion

### 4.1. Computational Complexity of MED and USD

MED and USD use quantum measurement methods for demodulation, the complexity of which is determined by the construction step of the POVM operator. Without loss of generality, with n users using quantum detection methods, 2n POVM operators are required for measurement with MED, and 2n+1 POVM operators are required for USD. The implementation steps for MED and USD are 2n and 2n+1, respectively. So, their computational complexity is O(n).

### 4.2. Comparison with NOMA

As is known, unlike OMA, the NOMA method uses non-orthogonal detection means such as POWER detection, which can enable multi-access without codecs, and usually the number of access users can be higher than OMA with lower detection complexity [12]. The MED method used in this paper uses a non-orthogonal coherent-state signal for detection and thus is essentially a NOMA method [34,35].

**Figure 8:**
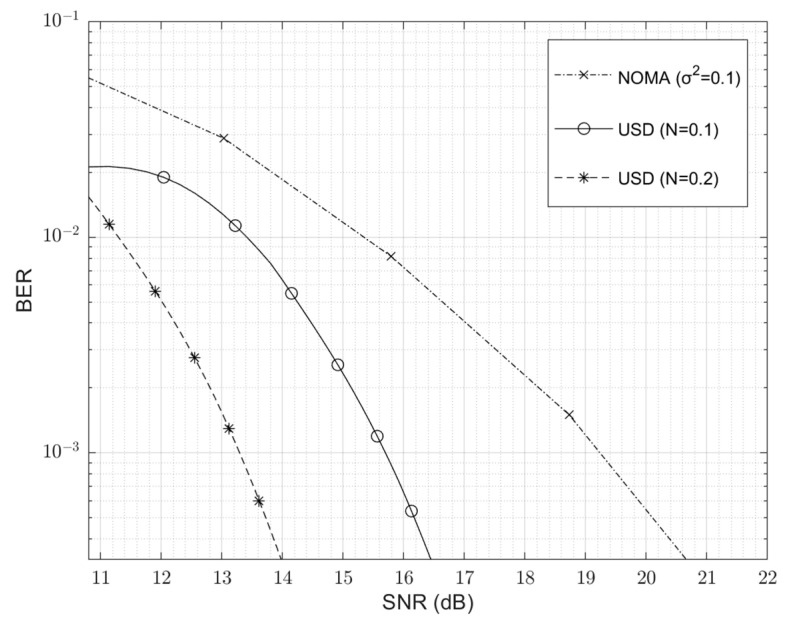
BER versus SNR performance of USD and NOMA.

The bigger difference is USD; although USD also uses non-orthogonal-state detection means and has many advantages over NOMA, it uses zero-error resolution measurements to achieve lower detection errors than traditional NOMA. In Figure 8, we compare the BER-SNR performance of USD and NOMA. The noise power of NOMA is set to 0.1 [12], and the noise of USD is set to 0.1 and 0.2, accordingly. From the results, we can see that USD has a better BER performance than NOMA, and unlike intuition, when the noise power increases, the BER of USD also decreases significantly. This is because its error probability, according to Equation (28), is not only determined by SNR, but also by the signal power (under a certain SNR, the noise power determines the signal power). In other words, when the signal power is large enough, a high BER performance can be guaranteed even with a small SNR. This characteristic of USD essentially comes from its ability to perform zero-error discrimination, extracting as much correct information as possible during detection, which is not available in traditional NOMA.

### 4.3. Does QAM Work for MED and USD?

**Figure 9:**
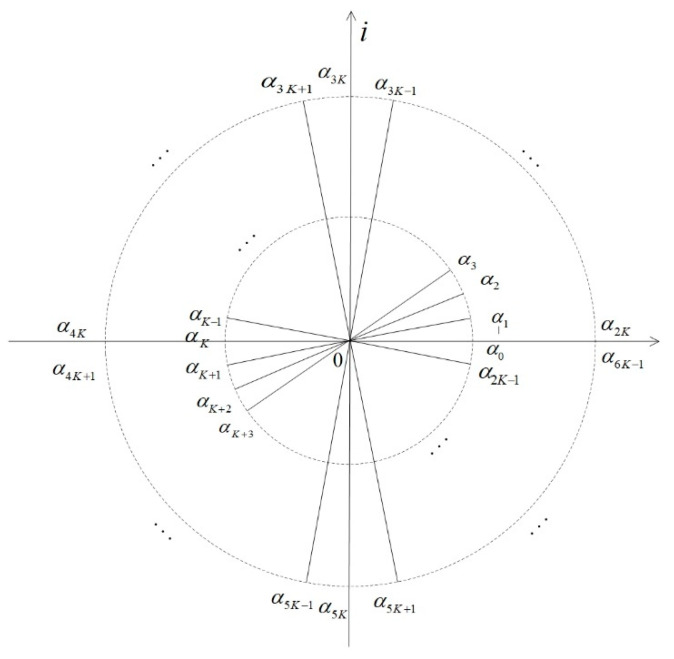
Coherent-state signals in the QAM form for 3K users.

The approach proposed in this paper targets M-PSK, which is easily associated with the possibility of considering the QAM approach. In fact, QAM uses amplitude and phase co-modulation, and a typical generalization of the coherent state form is shown in Figure 9, where the amplitude of 2K users in the outer ring is twice the amplitude of K users in the inner ring, i.e., $2\left|\alpha_{k<2K}\right|=\left|\alpha_{k\geq 2K}\right|$. Unfortunately, although multiple users can be represented by the quantum-state form of QAM, there is no good method for obtaining their optimal detection so far [35]. This is because the quantum state in the QAM form breaks the symmetry, which makes it very difficult to solve MED. The same tentative challenge is also true for USD [32].

This is really an area that has not been covered by our work, and we will be looking for possible solutions to this problem in the future.

## 5. Conclusions

In this paper, a quantum multi-access channel model based on symmetric coherent states is proposed, and an expression for the average inter-user interference is given. Based on this, the application of quantum detection in a multi-access channel with coherent-state signals is studied, and the channel capacities of MED and USD are given. The results show that MED is an optimal detection method that can reach the maximum channel capacity. The ideal USD measurement can discriminate between signals from different users with no errors, although its channel capacity is theoretically lower than that of MED, so USD is a better multi-user detection method than the MED measurement in scenarios where information error correction is considered.

Without loss of generality, we studied a two-user channel and examined its maximum capacity and inter-user detection error probability. Theoretical analysis and numerical results show that the maximum channel capacity of USD is smaller than that of MED and that there exists a region of channel capacity that is reachable by MED but not by USD. This verifies that USD sacrifices a portion of the channel capacity to achieve error-free discrimination. In addition, under the influence of thermal noise, the inter-user detection error probability of USD peaks at an average signal photon number of about 1.0 to 1.5 and decreases with increasing signal power. However, after the average photon number exceeds five, the USD can overcome the effect of noise by increasing the signal-to-noise ratio, showing the advantage in error correction. Additionally, we investigated the BER-SNR curves of the USD method compared to NOMA.

We have given theoretical results for general multi-user detection. In future work, we will focus on numerical simulations for the case of large-scale user numbers, as well as the study of large-scale user numbers in the noise case.

## Figures and Tables

**Table 1:** Definition of some useful notations and symbols in the text.

Notation or Symbol	Definition
BPSK	Binary phase shift keying
M-PSK	M-ary phase shift keying
QAM	Quadrature amplitude modulation
POVM	Operators of positive-operator-valued measure
K	Number of users in the multi-access channel
$\left|\alpha_{j}\right\rangle$	Coherent state
$E_{j}$	POVM operators
${\left|\mu \right|}^{2}$	Signal average photon number, characterize the power of the signal.
N	Thermal noise average photon number, characterize the power of the noise.
ρ	Density operator for signal superimposed thermal noise, usually $\rho ={\left(\pi N\right)}^{-1}\int exp\left(\frac{-{\left|\mu -\alpha \right|}^{2}}{N}\right)\left|\mu \right\rangle \left\langle \mu \right|d^{2}\mu$.
BER	Bit error rate
SNR	Signal-to-noise rate; in this paper, it is denoted as ${\left|\mu \right|}^{2}/N$.

## Data Availability

Not applicable.

## References

[1] Koonen T. (2018). Indoor Optical Wireless Systems: Technology, Trends, and Applications. J. Lightwave Technol..

[2] Gomez A., Shi K., Quintana C., Sato M., Faulkner G., Thomsen B.C., O’Brien D. (2015). Beyond 100-Gb/s Indoor Wide Field-of-View Optical Wireless Communications. IEEE Photonics Technol. Lett..

[3] Wang Y.Q., Huang X.X., Zhang J.W., Wang Y.G., Chi N. (2014). Enhanced performance of visible light communication employing 512-QAM N-SC-FDE and DD-LMS. Opt. Express.

[4] Kaushal H., Kaddoum G. (2017). Optical Communication in Space: Challenges and Mitigation Techniques. IEEE Commun. Surv. Tutor..

[5] Khalighi M.A., Uysal M. (2014). Survey on Free Space Optical Communication: A Communication Theory Perspective. IEEE Commun. Surv. Tutor..

[6] Messa A., Cossu G., Presi M., Schidl S., Schneider-Hornstein K., Zimmermann H., Ciaramella E. (2020). Detecting WDM visible light signals by a single multi-color photodiode with MIMO processing. Opt. Lett..

[7] He C.W., Cincotta S., Mohammed M.M.A., Armstrong J. (2019). Angular Diversity Aperture (ADA) Receivers for Indoor Multiple-Input Multiple-Output (MIMO) Visible Light Communications (VLC). IEEE Access.

[8] Fath T., Haas H. (2013). Performance Comparison of MIMO Techniques for Optical Wireless Communications in Indoor Environments. IEEE Trans. Commun..

[9] Jurado-Navas A., Raddo T.R., Garrido-Balsells J.M., Borges B.H.V., Olmos J.J.V., Monroy I.T. (2016). Hybrid optical CDMA-FSO communications network under spatially correlated gamma-gamma scintillation. Opt. Express.

[10] Li R.J., Dang A.H. (2017). A Novel Coherent OCDMA Scheme Over Atmospheric Turbulence Channels. IEEE Photonics Technol. Lett..

[11] Abouei J., Plataniotis K.N. (2012). Multiuser Diversity Scheduling in Free-Space Optical Communications. J. Lightwave Technol..

[12] Li R.J., Dang A.H. (2018). Multi-user access in wireless optical communication system. Opt. Express.

[13] Wang K., Kandeepan S., Alameh K. (2022). Filter-Enhanced Multi-User Scheme for Spatial Modulation Based Optical Wireless Communication Systems. J. Lightwave Technol..

[14] Helstrom C.W. (1969). Quantum detection and estimation theory. J. Stat. Phys..

[15] Ivanovic I.D. (1987). How to differentiate between non-orthogonal states. Phys. Lett. A.

[16] Dieks D. (1988). Overlap and distinguishability of quantum states. Phys. Lett. A.

[17] Wittmann C., Andersen U.L., Takeoka M., Sych D., Leuchs G. (2010). Demonstration of coherent-state discrimination using a displacement-controlled photon-number-resolving detector. Phys. Rev. Lett..

[18] Dolinar S.J. (1973). An optimum receiver for the binary coherent state quantum channel. Res. Lab. Electron. MIT Q. Prog. Rep..

[19] Cook R.L., Martin P.J., Geremia J.M. (2007). Optical coherent state discrimination using a closed-loop quantum measurement. Nature.

[20] Bondurant R.S. (1993). Near-quantum optimum receivers for the phase-quadrature coherent-state channel. Opt. Lett..

[21] Wittmann C., Takeoka M., Cassemiro K.N., Sasaki M., Leuchs G., Andersen U.L. (2008). Demonstration of near-optimal discrimination of optical coherent states. Phys. Rev. Lett..

[22] Tsujino K., Fukuda D., Fujii G., Inoue S., Fujiwara M., Takeoka M., Sasaki M. (2011). Quantum receiver beyond the standard quantum limit of coherent optical communication. Phys. Rev. Lett..

[23] Becerra F., Fan J., Baumgartner G., Polyakov S., Goldhar J., Kosloski J., Migdall A. (2011). M-ary-state phase-shift-keying discrimination below the homodyne limit. Phys. Rev. A.

[24] Becerra F., Fan J., Baumgartner G., Goldhar J., Kosloski J., Migdall A. (2013). Experimental demonstration of a receiver beating the standard quantum limit for multiple nonorthogonal state discrimination. Nat. Photonics.

[25] Banaszek K. (1999). Optimal receiver for quantum cryptography with two coherent states. Phys. Lett. A.

[26] Van Enk S. (2002). Unambiguous state discrimination of coherent states with linear optics: Application to quantum cryptography. Phys. Rev. A.

[27] Sedlák M., Ziman M., Přibyla O., Bužek V., Hillery M. (2007). Unambiguous identification of coherent states: Searching a quantum database. Phys. Rev. A.

[28] Bartůšková L., Černoch A., Soubusta J., Dušek M. (2008). Programmable discriminator of coherent states: Experimental realization. Phys. Rev. A.

[29] Waldherr G., Dada A.C., Neumann P., Jelezko F., Andersson E., Wrachtrup J. (2012). Distinguishing between Nonorthogonal Quantum States of a Single Nuclear Spin. Phys. Rev. Lett..

[30] Becerra F.E., Fan J., Migdall A. (2013). Implementation of generalized quantum measurements for unambiguous discrimination of multiple non-orthogonal coherent states. Nat. Commun..

[31] Mohseni M., Steinberg A.M., Bergou J.A. (2004). Optical Realization of Optimal Unambiguous Discrimination for Pure and Mixed Quantum States. Phys. Rev. Lett..

[32] Touzel M.A.P., Adamson R.B.A., Steinberg A.M. (2007). Optimal bounded-error strategies for projective measurements in nonorthogonal-state discrimination. Phys. Rev. A.

[33] Aboagye E.D., Chen S.-P. (2021). Deep space optical communications (DSOC) downlink simulation with varying PPM order. Opt. Quantum Electron..

[34] Elron N., Eldar Y.C. (2005). Quantum detection with uncertain states. Phys. Rev. A.

[35] Eldar Y.C., Megretski A., Verghese G.C. (2004). Optimal detection of symmetric mixed quantum states. IEEE Trans. Inf. Theory.

[36] Sasaki M., Hirota O. (1996). Optimum decision scheme with a unitary control process for binary quantum-state signals. Phys. Rev. A.

